# Efficacy and Safety of Durvalumab Combined with Daratumumab in Daratumumab-Refractory Multiple Myeloma Patients

**DOI:** 10.3390/cancers13102452

**Published:** 2021-05-18

**Authors:** Kristine A. Frerichs, Christie P. M. Verkleij, Meletios A. Dimopoulos, Jhon A. Marin Soto, Sonja Zweegman, Mary H. Young, Kathryn J. Newhall, Tuna Mutis, Niels W. C. J. van de Donk

**Affiliations:** 1 Cancer Center Amsterdam, Department of Hematology, Amsterdam UMC, Vrije Universiteit Amsterdam, 1081 HV Amsterdam, The Netherlands; k.frerichs@amsterdamumc.nl (K.A.F.); c.verkleij@amsterdamumc.nl (C.P.M.V.); j.marinsoto@amsterdamumc.nl (J.A.M.S.); s.zweegman@amsterdamumc.nl (S.Z.); t.mutis@amsterdamumc.nl (T.M.); 2 Department of Clinical Therapeutics, School of Medicine, National and Kapodistrian University of Athens, 11528 Athens, Greece; mdimop@med.uoa.gr; 3 Bristol Myers Squibb, Seattle, WA 98102, USA; mary.young@bms.com (M.H.Y.); katienewhall@comcast.net (K.J.N.)

**Keywords:** immunotherapy, multiple myeloma, PD-L1, CD38, daratumumab, checkpoint inhibitor, durvalumab

## Abstract

**Simple Summary:**

The CD38-targeting antibody daratumumab has marked activity in multiple myeloma through direct anti-tumor effects and immunomodulatory activity. However, eventually most patients will develop daratumumab-refractory disease. We hypothesized that daratumumab-resistance could be reversed by the addition of an inhibitor of the PD-1/PD-L1 signaling pathway, resulting in improved T- and NK-cell mediated anti-tumor immune responses. We therefore performed a phase 2 study to investigate the efficacy and safety of adding the PD-L1 checkpoint inhibitor durvalumab to daratumumab at the time of daratumumab failure. The toxicity profile of the daratumumab/durvalumab combination was acceptable, but none of the 18 enrolled patients achieved a clinical response. Immunomonitoring of bone marrow samples at baseline and during treatment showed a reduction of regulatory T-cell numbers and a decrease in the proportion of T-cells expressing LAG3 and CD8^+^ T-cells expressing TIM-3, whereas tumor cell characteristics were not affected. These results indicate that co-targeting PD-L1 at the time of daratumumab failure is insufficient to reverse daratumumab-resistance.

**Abstract:**

Daratumumab is active both as a single agent and in combination with other agents in multiple myeloma (MM) patients. However, the majority of patients will develop daratumumab-refractory disease, which carries a poor prognosis. Since daratumumab also has immunomodulatory effects, addition of the PD-L1 blocking antibody durvalumab at the time of progression may reverse daratumumab-resistance. The efficacy and safety of daratumumab and durvalumab in daratumumab-refractory relapsed/refractory MM patients was evaluated in this prospective, single-arm phase 2 study (NCT03000452). None of the 18 enrolled patients achieved PR or better. The frequency of serious adverse events was 38.9%, with one patient experiencing an immune related adverse event (grade 2 hyperthyroidism). No infusion-related reactions were observed. Analysis of tumor- and immune cell characteristics was performed on bone marrow samples obtained at baseline and during treatment. Daratumumab combined with durvalumab reduced the frequency of regulatory T-cells and decreased the proportion of T-cells expressing LAG3 and CD8^+^ T-cells expressing TIM-3, without altering T- and NK-cell frequencies. Durvalumab did not affect tumor cell characteristics associated with daratumumab resistance. In conclusion, the addition of durvalumab to daratumumab following development of daratumumab-resistance was associated with an acceptable toxicity profile, but was not effective. This indicates that inhibition of the PD-1/PD-L1 signaling pathway at the time of daratumumab-resistance is insufficient to reverse daratumumab-resistance.

## 1. Introduction

Daratumumab, a CD38-targeting monoclonal antibody for the treatment of multiple myeloma (MM), has potent single agent activity and a favorable toxicity profile [1,2]. Previous studies with daratumumab monotherapy reported overall response rates (ORR) ranging between 29.2 and 42.2% in relapsed/refractory (RR) MM patients with a median progression-free survival (PFS) ranging between 3.7 and 5.6 months [1,2,3]. The combination of daratumumab with immunomodulatory drugs (IMiD^®^ agents, Celgene, Summit, NJ, USA) and/or proteasome inhibitors (PIs) has improved response rates and PFS in both newly diagnosed MM and RRMM [4,5,6,7,8,9]. However, the prognosis of patients who become refractory to IMiD agents, PIs, and CD38-targeting monoclonal antibodies, such as daratumumab, remains poor, indicating an unmet medical need for new treatment options [10].

In addition to classic Fc-dependent immune effector functions, daratumumab has immunomodulatory effects by depleting CD38^+^ immune suppressive regulatory T-cells (Tregs), regulatory B-cells (Bregs) and myeloid derived suppressor cells (MDSCs), resulting in T-cell expansion and enhanced cytotoxic capacity of T-cells [11,12,13]. Therefore, combining daratumumab with modulators of other potent immune inhibitory pathways, such as the programmed-death 1 (PD-1)/programmed-death ligand 1 (PD-L1) axis, may further improve its efficacy or reverse resistance. PD-1 is upregulated on T-cells and NK-cells from MM patients, and MM cells have increased expression of its ligand, PD-L1, compared to healthy controls [14,15,16]. Importantly, we recently showed that blocking the PD-1/PD-L1 signaling pathway markedly improved anti-CD38 antibody-mediated anti-tumor activity in an anti-CD38 antibody naïve MM mouse model [15].

As monotherapy, the PD-1 inhibitors nivolumab and pembrolizumab did not induce objective responses in patients with RRMM. However, durable stable disease (SD) was observed in the majority of patients (57–63%) [17,18]. Although the evaluation of checkpoint inhibitors in combination with IMiD agents in MM has been halted because of an unfavorable benefit/risk profile of the PD-1 inhibitor pembrolizumab plus either lenalidomide-dexamethasone or pomalidomide-dexamethasone [19,20], several ongoing clinical studies are evaluating checkpoint inhibitors in combination with other drugs, such as monoclonal antibodies [21].

The monoclonal antibody durvalumab blocks PD-L1, resulting in an improved T-cell mediated anti-tumor response [22,23]. We hypothesized that the combination of daratumumab-induced immunomodulation and durvalumab-mediated immune checkpoint inhibition may result in improved T- and NK-cell mediated anti-tumor responses in MM patients, thereby overcoming daratumumab-resistance. We therefore evaluated the efficacy and safety of daratumumab and durvalumab in RRMM patients, who became refractory to daratumumab monotherapy or in combination with other anti-MM agents, in the FUSION-MM-005 study (NCT03000452). We also analyzed the impact of this combination on tumor cell characteristics and immune cell subsets.

## 2. Materials and Methods

### 2.1. Study Design

This was a prospective, single-arm, multicenter, open label phase 2 study to evaluate the efficacy and safety of daratumumab combined with durvalumab in daratumumab-refractory RRMM patients. The study consisted of two parts; part 1 had a two-stage design, and part 2 consisted of an expansion phase. In part 1 stage 1, the safety and tolerability profile of daratumumab and durvalumab was evaluated in 18 patients. If at least three patients achieved ≥ partial response (PR), 32 additional patients would be enrolled in part 1 stage 2 to evaluate safety and efficacy. If at least nine patients achieved ≥ PR upon completion of part 1, 70 additional patients would be enrolled in part 2 to confirm safety and efficacy data. The study was conducted in eight hospitals in Greece, Spain, Sweden and the United States. The study was approved by the central medical ethical committee (2016-003801-32) in each participating center and was conducted in accordance with the declaration of Helsinki. All patients provided written informed consent. The study was registered at ClinicalTrials.gov (accessed on 18 May 2021) as NCT03000452.

### 2.2. Study Objectives

The primary study objective was to determine the efficacy of daratumumab combined with durvalumab in daratumumab-refractory RRMM patients, based on the overall response rate (ORR) according to International Myeloma Working Group (IMWG) Uniform Response Criteria [24]. Secondary objectives were to determine time to response (TTR), duration of response (DOR), progression free survival (PFS), overall survival (OS), and safety of the combination treatment. In addition, changes in tumor characteristics, the microenvironment, and immune cell subsets were evaluated by explorative immune monitoring.

### 2.3. Study Population

Patients were eligible for enrollment in this study if they were 18 years or older, had RRMM with at least three prior lines of treatment, including a PI and an IMiD agent, or if they were double refractory to both a PI and an IMiD agent. Refractory disease was defined as no response to therapy (defined as failure to achieve minimal response [MR] or development of progressive disease [PD] while on treatment), or PD within 60 days of treatment discontinuation. Patients must have achieved MR or better to at least 1 prior treatment regimen. Furthermore, in their last line of therapy, all patients must have experienced disease progression during daratumumab treatment, either as monotherapy or in combination with other anti-MM agents. There was no daratumumab-free interval required. Patients were required to have measurable disease (serum M-protein ≥0.5 g/dL, urine M-protein ≥200 mg/24 hours or serum free light chain [FLC] ≥10 mg/dL and abnormal serum FLC ratio), toxicities from previous therapy resolved to ≤Grade 1, Eastern Cooperative Oncology Group (ECOG) Performance Status score ≤2, hemoglobin level ≥8 g/dL (≥4.9 mmol/L), platelet count ≥75 × 10^9^/L, absolute neutrophil count ≥1.0 × 10^9^/L, creatinine clearance ≥45 mL/min, serum calcium ≤13.5 mg/dL (≤3.4 mmol/L), serum aspartate aminotransferase (AST) or alanine aminotransferase (ALT) ≤2.5× upper limit of normal (ULN), and serum total bilirubin ≤1.5× ULN. Patients had to agree to use contraception. Exclusion criteria included prior treatment with anti-CTLA-4, anti-PD-1 or anti-PD-L1 monoclonal antibodies, cancer vaccines, an autologous stem cell transplantation within 12 weeks prior to enrollment, and/or an allogeneic stem cell transplantation at any time. In addition, patients with clinically relevant active comorbidities, a history of autoimmune disorders, inflammatory disorders, or malignancy within the last 5 years were excluded.

### 2.4. Treatment

Patients received daratumumab treatment (16 mg/kg, intravenous infusion) according to the approved schedule (cycle 1 and 2: on days 1, 8, 15, and 22; cycles 3–6: on days 1 and 15; thereafter on day 1 of each 28-day treatment cycle). Daratumumab dosing was continued according to the number of infusions administered during the prior daratumumab-containing regimen, without re-intensification. Patients received intravenous durvalumab at a flat dose of 1500 mg on day 2 of cycle 1, and on day 1 of each subsequent 28-day treatment cycle. If daratumumab and durvalumab were administered on the same day, daratumumab was administered first. Study treatment was continued until PD or unacceptable toxicity. To prevent infusion related reactions, pre- and post-infusion medication was administered according to the approved schedule [2]: methylprednisolone (100 mg; following the second infusion the dose could be reduced to 60 mg), acetaminophen (650–1000 mg) and diphenhydramine (25–50 mg) were administered prior to every daratumumab administration. Montelukast was given prior to the first administration. In addition, methylprednisolone (20 mg) was administered on the 2 days following each administration of daratumumab. Furthermore, all patients received herpes zoster prophylaxis (acyclovir 800 mg twice daily, or equivalent).

### 2.5. Safety and Efficacy Assessments

Adverse events (AEs) were graded according to the National Cancer Institute Common Terminology Criteria for Adverse Events (NCI CTCAE) version 5.0 [25]. Safety assessments were performed from the first date of administration of either study drug until 90 days after the last dose of either study drug. In addition, AEs that occurred beyond this timeframe, but were assessed by the treating physician as related to the study drug, were also considered treatment-emergent adverse events (TEAE). Treatment response was assessed at the end of each cycle according to the IMWG Uniform Response Criteria [24]. Accordingly, stable disease (SD) was defined as not meeting criteria for complete response (CR), very good partial response (VGPR), partial response (PR) or progressive disease (PD) [24].

### 2.6. Flow Cytometric Analysis of Tumor- and Immune Cell Characteristics

Bone marrow (BM) aspirates were obtained at baseline (prior to treatment with daratumumab and durvalumab) and after 6 weeks of treatment (cycle 2 day 15 or C2D15). BM mononuclear cells (MNCs) from these BM aspirates were isolated by Ficoll-Hypaque density-gradient centrifugation, and cryopreserved until analysis. In these BM samples, we characterized tumor cells, T-cells, Tregs, B-cells, Bregs, NK-cells and MDSCs by staining 4 × 10^6^ nucleated cells with CD45 Krome Orange (Beckman Coulter, Brea, CA, USA), HuMax-003 FITC (Genmab/Janssen), CD138 BUV395, CD56 BUV737, CD3 BUV395, CD4 PerCP, CD8 BUV737 (all Becton Dickinson, Franklin Lakes, NJ, USA), CD25 PE (Biolegend, San Diego, CA, USA), CD127 PE-CF594, CD19 PE-CF594, CD24 PE, CD16 BV421, CD14 PE (all Becton Dickinson), CD11b PerCP-Cy5.5 (Biolegend, San Diego, CA, USA) and CD33 PC7 (Beckman Coulter, Brea, CA, USA). We used CD45RA AlexaFluor 700 and CD197 (C-C chemokine receptor type 7 [CCR7]) BV786 (both Becton Dickinson) to determine the proportion of T-cell memory subsets. Cells were stained with Human Leukocyte Antigen–DR isotype (HLA-DR) APC-H7 (Becton Dickinson) to evaluate T-cell activation, and with CD152 (cytotoxic T-lymphocyte-associated protein 4 [CTLA-4]) PE-CY7 (both Biolegend), CD47 BV605, CD270 (Herpesvirus entry mediator [HVEM]) BV711, CD274 (PD-L1) BV786 clone MIH1 (this antibody binds to a PD-L1 epitope distinct from the epitope bound by durvalumab, and allows detection of PD-L1 during durvalumab treatment [26]), CD279 (PD-1) BV711, CD223 (Lymphocyte-activation gene 3 [LAG3]) APC, CD272 (B- and T-lymphocyte attenuator [BTLA]) BV421, CD366 (T-cell immunoglobulin and mucin-domain containing-3 [TIM-3]) BV605 (all Becton Dickinson, Franklin Lakes, NJ, USA) to assess the expression of immune checkpoints. CD59 BV421 (Becton Dickinson, Franklin Lakes, NJ, USA) and CD55 PE-CY7 (Biolegend) were used to determine the expression of complement inhibitors. The LIVE/DEATH Fixable Death cell staining kit (Invitrogen, Carlsbad, CA, USA) was used to determine sample viability prior to cytoplasmic staining for immunoglobulin light chains using anti-kappa PE (DAKO) and anti-lambda APC-H7 (Becton Dickinson). To assess CD38 expression on tumor and effector cells irrespective of daratumumab treatment, we used HuMax-003 FITC, which binds to a CD38 epitope distinct from the epitope recognized by daratumumab [27].

MM cells are defined as viable CD45^dim^CD38^+^, kappa^+^ or lambda^+^ cells, after exclusion of CD45^high^ lymphocytes and CD19^+^CD56^−^ cells. As previously described, Tregs were defined as CD3^+^CD4^+^CD127^dim^CD25^+^ [12], Bregs as CD19^+^CD24^+^CD38^+^ [12], and MDSCs as CD11b^+^CD33^+^CD14^−/+^HLA-DR^−^ [28]. Naïve T-cells were defined as CD45RA^+^CCR7^+^, central memory (CM) T-cells as CD45RA^−^CCR7^+^, effector memory (EM) T-cells as CD45RA^−^CCR7^−^, and terminally differentiated effector memory T-cells expressing CD45RA (TEMRA) as CD45RA^+^CCR7^−^ [29].

Flow cytometry was performed using a 7-laser LSRFORTESSA (Becton Dickinson). Fluorescent labeled beads (CS&T beads, Becton Dickinson) were used daily to monitor the performance of the flow cytometer and verify optical path and stream flow. This procedure enables controlled standardized results and allows the determination of long-term drifts and incidental changes within the flow cytometer. No changes were observed which affected the results. Compensation beads were used to determine spectral overlap, compensation was automatically calculated using FACSDiva software (BD Biosciences, San Jose, CA, USA). Flow cytometry data were analyzed using FCS express software, version 6 (DeNovo software, Pasadena, CA, USA).

### 2.7. RNA Sequencing

RNA-seq was performed on whole BM, not on purified MM cells. RNA from 14 BM samples obtained at baseline and from five samples obtained at cycle 2 day 15, was extracted using the RNeasy Micro kit (Qiagen, Venlo, the Netherlands). Quality was controlled using the spectrophotometric measurements and agarose gel analysis. RNA sequencing (RNA-seq) libraries were produced using polyA enrichment and strand-specific RNA library construction with barcodes. Samples were sequenced on the HiSeq 2500 system (Illumina, San Diego, CA, USA) with 2 × 50 bp read lengths using truSeq SBS v4 chemistry (Illumina, San Diego, CA, USA). Subsequently, RNA-seq reads were mapped to the reference human genome and the parameters were set to count read number per gene while mapping. RNA-sequencing count data were normalized using trimmed mean of M values (TMM) and transformed to log2 counts per million along with observation level weights using voomWithQualityWeights from the limma R package [30]. Genes with a TMM count of at least 1 in 10% of libraries, resulting in almost 19,000 genes, were kept for further analysis. To find differentially expressed genes between screening and cycle 2 day 15, a linear model with a random effect for individual was run within each cohort. Finally, visualization of the results was done with GSVA (gene set variation analysis) to calculate a summary metric for each gene set.

### 2.8. Statistics

Statistical analyses were performed according to the intention-to-treat (ITT) principle. PFS was calculated from day 1 of treatment until progression or death, whichever came first. OS was measured from day 1 of treatment until death from any cause. Patients still alive at the date of last follow-up were censored. PFS and OS were estimated using the Kaplan-Meier method. Safety analyses were performed in all patients who received at least 1 dose of daratumumab plus durvalumab.

Comparisons between continuous variables were performed using Mann-Whitney U test or Wilcoxon matched-pairs signed rank test. Statistical analyses were performed in GraphPad Prism (version 7, San Diego, CA, USA) and SPSS Statistics (version 24, IBM, Armonk, NY, USA); *p*-values below 0.05 were considered significant. For analyses of RNA-seq data, *p*-values were corrected for multiple testing by using the Benjamini-Hochberg false discovery rate (FDR) method.

## 3. Results

### 3.1. Efficacy of Daratumumab and Durvalumab in Daratumumab-Refractory RRMM Patients

In part 1 stage 1 of the study, 18 patients were included, with a median of five prior lines of treatment (range 3–16; Table 1). Directly preceding enrollment, all patients had developed disease progression during a daratumumab-containing regimen, which consisted of daratumumab monotherapy in five patients (27.8%), daratumumab combined with a proteasome inhibitor in three patients (16.7%; bortezomib *n* = 1, carfilzomib *n* = 2) and combined with an IMiD agent in 10 patients (55.6%; lenalidomide *n* = 3, pomalidomide *n* = 7). Response (≥PR) to prior daratumumab–based therapy was 55.6%, and the median duration of prior daratumumab treatment was 278 days (range 48–700). Importantly, two patients (11.1%) had already failed two daratumumab-containing treatment regimens prior to study enrollment. The median interval between last daratumumab administration and study entry was 48 days (range 7–183).

**Figure 1:**
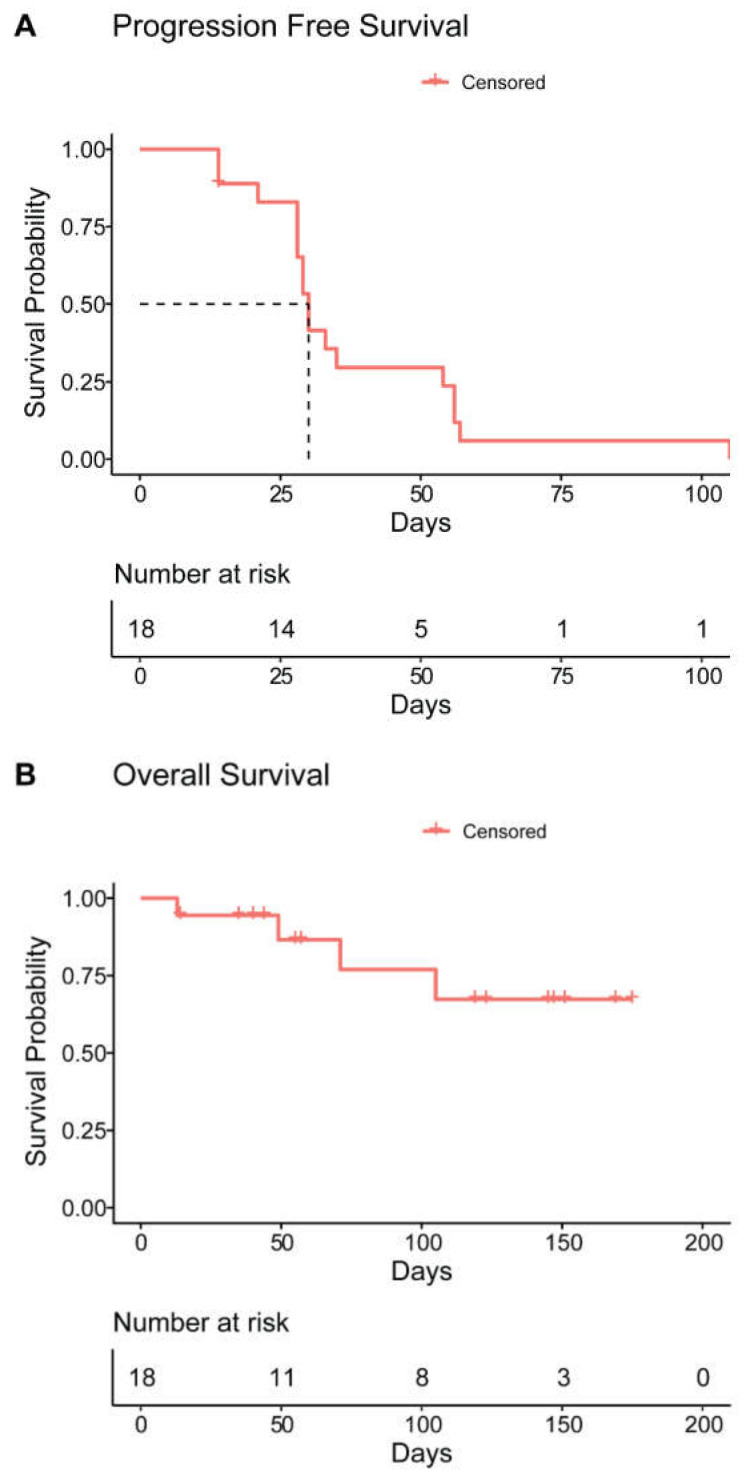
(**A**) Progression free survival (PFS) and (**B**) overall survival (OS) for patients treated with daratumumab and durvalumab. PFS and OS were estimated using the Kaplan-Meier method.

None of the 18 patients achieved PR or better following treatment with daratumumab combined with durvalumab. Best response was SD in eight patients (44.4%), nine patients (50%) developed PD immediately after the first cycle, and one patient (5.6%) was not evaluable for response. For the patients who achieved SD, the median duration of disease stabilization was 55 days (range 21–105). With a median duration of follow up in surviving patients of 88 days (range 14–175), the median PFS was 30 days (95% CI 28–32; Figure 1A). The median OS was not reached (Figure 1B). Although subgroups are small, type of prior therapy had no significant impact on PFS.

As per protocol and based on these results, part 1 stage 2 was not initiated, and the study was closed for further enrollment.

### 3.2. Safety of Daratumumab and Durvalumab

All patients experienced at least 1 AE from the first dose until 90 days after the last administration of either one of the study drugs, and the median number of AEs per patient was 8 (range 1–21; Table 2). Eleven SAEs were reported in seven patients (38.9%): worsening of general condition due to disease progression (*n* = 6), acute kidney failure (*n* = 1), fever (*n* = 1), heart failure (*n* = 1), left pleural effusion (*n* = 1) and pneumonia (*n* = 1). No daratumumab or durvalumab-related infusion reactions were reported. One patient experienced an immune-related AE (grade 2 hyperthyroidism), which resolved upon initiation of thyroid suppression therapy. The all-cause mortality was 22.2% (*n* = 4), and in all four patients the cause of death was disease progression. None of the other adverse events were fatal.

### 3.3. Flow Cytometric Analysis of Tumor- and Immune Cell Characteristics

Since we hypothesized that addition of durvalumab could improve the immunomodulatory effects of daratumumab, resulting in reduced immune suppression and enhanced T- and NK-cell mediated anti-tumor responses, we performed immune monitoring studies in BM samples obtained at baseline (daratumumab-refractory disease, prior to adding durvalumab; *n* = 17) and after 6 weeks of treatment with daratumumab combined with durvalumab (C2D15; *n* = 8). We assessed tumor cell characteristics associated with immune suppression (PD-L1, CD47, and HVEM) or known tumor features associated with response/resistance to daratumumab treatment (expression levels of CD38 and the complement inhibitory proteins CD55 and CD59). We also assessed the frequencies and characteristics of immune cells. Gating strategies for different immune cells are shown in Figure S1.

**Figure 2:**
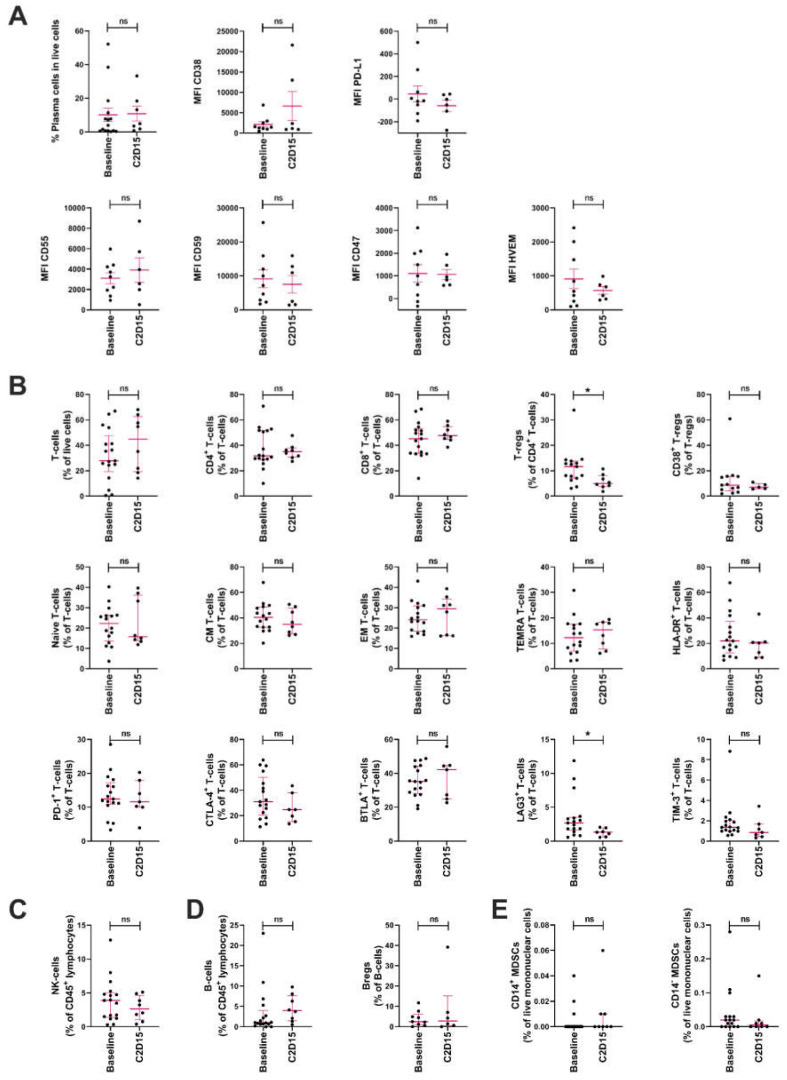
Effect of durvalumab and daratumumab treatment on the frequencies and characteristics of tumor cells and immune cells. Frequencies and characteristics of tumor cells and immune cells were assessed by flow cytometry in BM samples obtained from patients at study entry (baseline; *n* = 17) and after 6 weeks of treatment with daratumumab and durvalumab (C2D15; *n* = 8). (**A**) The frequency of plasma cells, as well as their cell surface expression of CD38, PD-L1, CD55, CD59, CD47 and HVEM. (**B**) The frequency of T-cells and T-cell subsets (CD4^+^ T-cells; CD8^+^ T-cells; Tregs; CD38^+^ Tregs; naïve T-cells; CM T-cells; EM T-cells; TEMRA T-cells; HLA-DR^+^ T-cells; PD-1^+^ T-cells; CTLA-4^+^ T-cells; BTLA+ T-cells; LAG3^+^ T-cells; and TIM-3^+^ T-cells). (**C**) The frequency of NK-cells. (**D**) The frequency of B-cells and Bregs. (**E**) The frequency of CD14^+^ and CD14^−^ MDSCs. Dots represent individual data, lines represent median value and error bars represent interquartile range. Differences between baseline and C2D15 were assessed using Mann-Whitney U-tests; * *p* < 0.05. Abbreviations: ns, not significant; PD-L1, programmed death-ligand 1; HVEM, Herpesvirus entry mediator; Tregs, regulatory T-cells; CM, central memory; EM, effector memory; TEMRA, terminally differentiated effector memory T-cells expressing CD45RA; HLA-DR, human leukocyte antigen DR isotype; PD-1; programmed death receptor 1; CTLA-4, cytotoxic T-lymphocyte associated protein 4; BTLA, B- and T-lymphocyte attenuator; LAG3, lymphocyte-activation gene 3; TIM-3, T-cell immunoglobulin and mucin domain 3; Bregs, regulatory B-cells; MDSCs, myeloid derived suppressor cells.

The median frequency of PCs in these BM aspirates remained unchanged upon addition of durvalumab to daratumumab (Figure 2A). As expected, due to prior daratumumab treatment, baseline CD38 expression on tumor cells was low [31]. The addition of durvalumab to daratumumab did not further affect CD38 expression levels. Similarly, cell surface expression of complement inhibitory proteins CD55 and CD59, and the immune inhibitory proteins PD-L1, CD47 and HVEM, were not affected by treatment with daratumumab and durvalumab (Figure 2A). In order to assess PD-L1 expression, we used a flow antibody that binds to a different epitope compared with durvalumab. This excluded the possibility that binding of durvalumab masked the detection of PD-L1. Although subgroups are small, type of prior therapy had no significant impact on baseline PD-L1 expression on tumor cells.

Daratumumab has been shown to reduce the frequency of CD38^+^ Tregs [12]. We observed that, upon the addition of durvalumab to daratumumab, total Treg counts were significantly reduced (baseline median 11.7%; C2D15 5.0%; *p* = 0.02), while the frequency of CD38^+^ Tregs was not further affected by adding durvalumab (*p* = 0.775; Figure 2B). In addition, frequencies of T-cells, frequencies of CD4^+^ and CD8^+^ T-cell subsets, frequencies of differentiation subsets (naïve, CM, EM and TEMRA T-cells), frequencies of activated T-cells (based on HLA-DR expression), and the frequency of T-cells expressing the immune checkpoint proteins PD-1, CTLA-4, TIM-3 or BTLA, were not affected by combination treatment (Figure 2B). However, a reduction in the frequency of T-cells expressing the immune checkpoint LAG3 was observed after the addition of durvalumab (baseline median 2.68%; C2D15 1.31%; *p* = 0.0244; Figure 2B). Similar results were obtained when CD4^+^ and CD8^+^ T-cell subpopulations were analyzed separately. In addition, we observed a significant reduction in the frequency of CD4^+^ T-cells with a central memory phenotype, and of CD8^+^ T-cells expressing TIM-3 at C2D15 (Figure S2A,B). Type of prior therapy had no significant impact on the proportion of PD-1^+^ CD4^+^ and CD8^+^ T-cells at the time of enrollment into the study.

As expected, patients had low NK-cell levels at baseline as a result of their prior daratumumab exposure [27,32]. During treatment with daratumumab and durvalumab, the frequency of NK-cells was not significantly affected (Figure 2C). Daratumumab has also been shown to reduce the frequency of Bregs [12]. Combination treatment with daratumumab and durvalumab did not have an additional impact on the frequencies of Bregs, and the frequency of B-cells was also not altered (Figure 2D). Finally, since MDSCs have been shown to suppress the T-cell mediated anti-tumor response in MM [28], we analyzed CD14^+^ and CD14^−^ MDSCs in these BM samples. The frequencies of these MDSCs were not affected by treatment with daratumumab and durvalumab (Figure 2E).

### 3.4. RNA Sequencing of BM Samples

**Figure 3:**
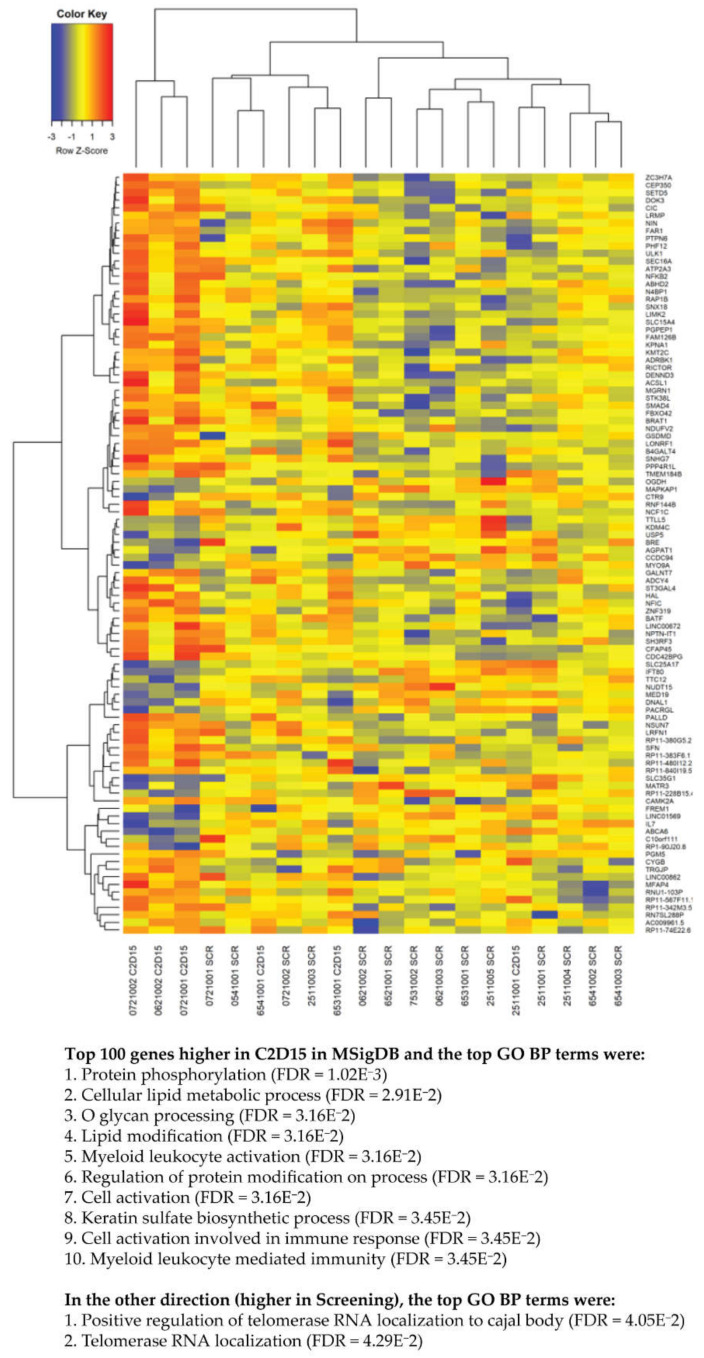
Comparison of the transcriptome of BM cells obtained at baseline and after initiation of daratumumab/durvalumab treatment. Shown are the top 100 differentially expressed genes between baseline samples (*n* = 14) and those obtained after 6 weeks of daratumumab/durvalumab treatment (*n* = 5). To perform gene set enrichment, the top genes were entered into the Broad’s MSigDB website, which computed the overlap with GO term gene sets. *p*-values were corrected for multiple testing by using the Benjamini-Hochberg false discovery rate (FDR) method.

We also compared the transcriptome of unsorted BM cells obtained at baseline (*n* = 14 samples) with BM cells obtained after 6 weeks of daratumumab/durvalumab treatment (*n* = 5 samples). Figure 3 shows the top 100 differentially expressed genes between baseline samples and those obtained after 6 weeks of daratumumab/durvalumab treatment. Hierarchical clustering shows that individual patient samples at baseline and cycle 2 day 15 separate mostly based on the time point that the sample was obtained. This suggests that there were common effects of the treatment on gene expression. To perform gene set enrichment, the top genes were entered into the Broad’s MSigDB website, which computed the overlap with GO term gene sets. The top genes that were upregulated at cycle 2 day 15 as compared to baseline samples, were classified as being related to protein and lipid modification, lipid metabolomics, and immune response, including myeloid leucocyte activation, cell activation involved in immune response, and myeloid leucocyte-mediated immunity. Genes that were higher at baseline as compared to cycle 2 day 15 were classified according to GO enrichment analysis as belonging to regulation of telomerase localization.

RNA-seq analysis of BM cells showed no significant difference in expression of genes encoding immune checkpoint proteins (e.g., *PCDC1* (encoding PD-1), *BTLA*, *LAG3*, *HAVCR2* (encoding TIM-3), *CD274* (encoding PD-L1), and *PDCD1LG2* (encoding PD-L2) between baseline and after 6 weeks of treatment. Furthermore, there was no difference in gene expression of cytokines (*IFNG*), chemokines (*CXCL9*, *CXCL10*), chemokine receptors (*CCR7*), cytokines receptors (*IL2RA*, *IL7R*), activation markers (*HLA-DR*) and cytotoxic molecules (*GzmA* and *GzmB*) between both groups. In addition, expression of transcription factors affected by or regulating expression of interferon-γ (*EOMES*, *TBX21*, and *STAT1*) was not significantly altered. In line with the flow cytometry results showing no change in MM cell frequency, also expression of MM-associated genes such as *SDC1* (CD138), *NCAM1* (CD56) and *CD38* remained unchanged.

## 4. Discussion

Daratumumab is increasingly used for the treatment of both newly diagnosed and relapsed/refractory MM patients. Resistance to daratumumab is associated with a poor survival, indicating an unmet need for new treatment options in daratumumab-refractory MM. In this study, we show that the strategy of adding durvalumab to daratumumab at the time of daratumumab failure is not effective. Although the frequency of Tregs in the BM microenvironment was reduced, and proportion of T-cells expressing LAG3 decreased upon combination treatment with daratumumab and durvalumab, we observed no changes in frequencies of T- or NK-cells. This indicates that blockade of the PD-1/PD-L1 signaling pathway alone is insufficient to reverse resistance to daratumumab in heavily pretreated MM patients.

While the combination of daratumumab and durvalumab did not induce clinical responses, the combination had a manageable toxicity profile, without patients discontinuing treatment due to AEs, and without treatment-related mortality. The rate of any grade infections was slightly higher compared to daratumumab monotherapy [1,2]. There were no daratumumab or durvalumab-related infusion reactions, and only 1 patient presented with an immune-related AE (hyperthyroidism), which resolved upon treatment.

We hypothesized that the combined immunomodulatory effects of daratumumab and durvalumab would improve T- and NK-cell mediated anti-tumor immune responses and could overcome daratumumab resistance. Indeed, mouse models have previously shown improved anti-tumor activity when a CD38-targeting antibody is combined with PD-1 neutralizing antibody in a CD38-targeting antibody naïve setting [15,33]. Here, we show that addition of durvalumab to daratumumab resulted in a significant reduction in the frequency of Tregs. Several studies have shown that in MM, Tregs are increased in numbers, and that these cells play an important role in the suppression of T-cell effector function, resulting in a reduced T-cell mediated anti-tumor response and impaired survival [28,34,35,36]. PD-1/PD-L1 interactions not only directly cause dysfunction and exhaustion of effector T-cells, but also suppress T-cell function indirectly by promoting Treg development [37,38,39,40]. Indeed, several studies showed that blockade of the PD-1/PD-L1 signaling pathway limits Treg survival and reduces the frequency of Tregs, which may explain the observed Treg reduction in our study [39,41,42,43,44]. Durvalumab also reduced the proportion of T-cells expressing LAG3 and CD8^+^ T-cells expressing TIM-3 in our daratumumab-refractory patients. Expansion of exhausted T-cells with upregulation of LAG3 and TIGIT has previously been described in patients resistant to daratumumab combined with pomalidomide-dexamethasone [45]. The frequencies of other immune cell subsets, or the proportion of T-cells expressing other inhibitory checkpoint proteins remained unchanged.

RNA-seq analysis of unsorted BM cells showed that expression of genes encoding immune checkpoint proteins remained unchanged. However, the number of samples for this evaluation was limited and involved analysis of unseparated bone marrow cell populations. Therefore, we were unable to assess mutational load in purified MM cells and correlate mutational load with response to treatment or changes in immune cell composition. Notably, tumor mutational burden is a genomic biomarker that predicts response to immune checkpoint inhibitors in other types of cancer [46,47].

We cannot rule out that pre- and post-infusion corticosteroid administration had a negative impact on T-cell immunity in our study. In patients with non-small-cell lung cancer, who were treated with PD-(L)1 inhibitors, baseline corticosteroid use of ≥10 mg of prednisone or equivalent was associated with poorer outcome, although it remains unclear whether baseline corticosteroids represent correlation (e.g., patients with aggressive disease) or causation [48]. Importantly, preliminary results show that rapid tapering of corticosteroids is feasible in MM patients, who receive subcutaneous daratumumab, without an increase in infusion related reactions [49]. These data help to guide future daratumumab/PD-(L)1 inhibitor combination regimens, where limited use of corticosteroids should be considered.

Although the mechanisms of resistance to daratumumab treatment remain to be fully elucidated, reduced baseline expression of CD38 on the tumor cell surface is associated with a lower response rate to daratumumab monotherapy, probably through diminished activity of Fc-receptor-dependent effector mechanisms such as complement-dependent cytotoxicity (CDC) and antibody-dependent cellular cytotoxicity (ADCC). In addition, CD38 expression is rapidly reduced during daratumumab treatment, further reducing induction of ADCC and CDC. Increased expression of the complement inhibitors, CD55 and CD59, has also been shown to contribute to development of daratumumab resistance [31,50]. As expected, prior to the addition of durvalumab to daratumumab, the MM cells had low cell surface levels of CD38 due to prior daratumumab treatment. However, the addition of durvalumab to daratumumab had no additional impact on CD38 expression levels and did not lower CD55 and CD59 expression. This suggests that durvalumab is not able to restore ADCC or CDC in daratumumab-refractory patients. Furthermore, upregulation of other immune inhibitory molecules such as TIGIT on T-cells has also been shown to contribute to the development of resistance to daratumumab combined with pomalidomide-dexamethasone [45]. This may suggests that inhibition of the PD-1/PD-L1 pathway alone is not sufficient to restore sensitivity to daratumumab.

A limitation of this study is that BM samples cannot be compared with BM samples obtained prior to initiation of daratumumab treatment. Further studies are needed to compare daratumumab-naïve and -refractory BM samples in order to analyze daratumumab-induced tumor and immune cell alterations, which contribute to daratumumab-resistance.

Optimal durvalumab-mediated immunomodulation probably requires more time than the median duration of treatment of 8 weeks in this study, which may also partially explain the lack of response in our study. Therefore, several ongoing studies (NCT03184194; NCT01592370; NCT02431208), which evaluate the combination of PD-1 or PD-L1 inhibitors with daratumumab in daratumumab-naïve RRMM patients, will provide further insight in clinical efficacy and immunomodulatory changes induced by combination treatment. The direct combination of daratumumab with PD-(L)1 inhibitors may lead to an improved response rate, compared to adding PD-(L)1 inhibitors at the same time of development of daratumumab resistance.

## 5. Conclusions

In conclusion, the addition of durvalumab to daratumumab in daratumumab-refractory RRMM resulted in a reduced frequency of BM-localized Tregs and decrease in T-cells expressing LAG3, but this was not sufficient to abrogate daratumumab-resistance. Since the daratumumab and durvalumab combination was safe, further evaluation of the combined immunomodulatory effects is warranted in daratumumab-naïve RRMM patients.

## Figures and Tables

**Table 1:** Baseline characteristics.

Characteristic	Daratumumab and Durvalumab
	*n* = 18
Age, years; median (range)	65.5 (40–75)
Male sex, *n* (%)	7 (38.9)
ECOG performance status, *n* (%)	
- 0	12 (66.7)
- 1	5 (27.8)
- 2	1 (5.6)
- 3	0
ISS stage at entry, *n* (%)	
- Stage I	1 (5.6)
- Stage II	5 (27.8)
- Stage III	4 (22.2)
- Unknown	8 (44.4)
M-protein, *n* (%)	
- IgA kappa	4 (22.2)
- IgG kappa	9 (50.0)
- IgG lambda	3 (16.7)
- FLC lambda	2 (11.1)
FISH analysis on isolated plasma cells, *n* (%)	8 (44.4)
High risk cytogenetic abnormalities, *n* (%)	
- t(4;14)	Unknown
- del(17p)	2 (25)
- t(14;16)	Unknown
- amp(1q)	1 (12.5)
- del(13q)	2 (25)
Prior lines of treatment, median (range)	5 (3–16)
Prior IMiD agent, *n* (%)	
- Thalidomide	5 (27.8)
- Lenalidomide	18 (100)
- Pomalidomide	14 (77.8)
Prior PI, *n* (%)	
- Bortezomib	18 (100)
- Carfilzomib	8 (44.4)
- Ixazomib	0
Prior alkylating agents, *n* (%)	
- Cyclophosphamide	12 (66.7)
- Melphalan	15 (83.3)
Prior monoclonal antibodies, *n* (%)	
- Daratumumab	18 (100)
- Elotuzumab	1 (5.6)
Prior autologous stem cell transplantation, *n* (%)	15 (83.3)
Most recent daratumumab-containing regimen, *n* (%)	
- Daratumumab monotherapy	5
- Daratumumab + PI	3
- Daratumumab + IMiD agent	10
Best response to prior daratumumab-containing regimen, *n* (%)	
- CR	0
- VGPR	3 (16.7)
- PR	7 (38.9)
- MR	2 (11.1)
- SD	3 (16.7)
- PD	2 (11.1)
- Unknown	1 (5.6)
Creatinine clearance at entry, *n* (%)	
- ≥60 mL/min	14 (77.8)
- 30–60 mL/min	3 (16.7)
- <30 mL/min	1 (5.6)
Platelet count at entry, *n* (%)	
- ≥150 × 10^9^/L	13 (72.2)
- <150 × 10^9^/L	5 (27.8)
LDH at entry, *n* (%)	
- Normal	14 (77.8)
- Elevated	4 (22.2)

Abbreviations: ECOG, Eastern Cooperative Oncology Group; ISS, International Staging System; IMiD, immunomodulatory drug; PI, proteasome inhibitor; CR, complete remission; VGPR, very good partial response; PR, partial response; MR, minimal response; SD, stable disease; PD, progressive disease.

**Table 2:** Adverse events occurring in more than 1 patient.

Adverse Event Term	Any Grade, *n* (%)	Grade ≥ 3, *n* (%)
Hematological toxicity		
- Anemia	13 (72.2)	8 (44.4)
- Thrombocytopenia	7 (38.9)	4 (22.2)
- Neutropenia	5 (27.8)	4 (22.2)
Infections	6 (33.3)	1 (5.6)
- Common cold	2 (11.1)	0
- Herpes zoster	1 (5.6)	0
- URTI	1 (5.6)	0
- Pneumonia	2 (11.1)	1 (5.6)
Anorexia	2 (11.1)	0
Back pain	2 (11.1)	0
Bone pain	5 (27.8)	0
Dyspnea	4 (22.2)	0
Edema	2 (11.1)	0
Fatigue	11 (61.1)	1 (5.6)
Fever	3 (16.7)	0
Hypercalcemia	3 (16.7)	0
Hypertension	2 (11.1)	1 (5.6)
Hyperuricemia	3 (16.7)	2 (11.1)
Nausea	2 (11.1)	0
Neuralgia	2 (11.1)	0
Pain extremities	3 (16.7)	0
Renal failure *	8 (44.4)	3 (16.7)
Weight loss	3 (16.7)	0
General deterioration due to disease progression	6 (33.3)	4 (22.2) †

* Renal failure encompasses acute kidney injury, acute renal failure and increased creatinine levels. † 4 patients died as a result of general deterioration due to disease progression, CTC grade 5. Abbreviations: URTI, upper respiratory tract infection.

## Data Availability

The data presented in this study are available on request from the corresponding author. The data are not publicly available due to contractual restrictions.

## Supplementary Materials

The following is available online at https://www.mdpi.com/article/10.3390/cancers13102452/s1, Figure S1: Gating strategy in flow cytometric analyses, Figure S2: Effect of daratumumab and durvalumab on the frequencies and characteristics of CD4^+^ and CD8^+^ T-cell subsets.
