# Supplementary material for: Efficacy and Safety of Durvalumab Combined with Daratumumab in Daratumumab-Refractory Multiple Myeloma Patients
Source: Cancers (Basel). 2021 May 18;13(10):2452. doi: 10.3390/cancers13102452 (PMC8158123; doi:10.3390/cancers13102452)
Supplement: Supplementary file 1 [file cancers-13-02452-s001.zip › cancers-1186351-supplementary.pdf]

cancers

MDPI

Supplementary Materials

Efficacy and safety of durvalumab combined with daratumumab in daratumumab-refractory multiple myeloma patients

Kristine A. Frerichs, Christie P.M. Verkleij, Meletios A. Dimopoulos, Jhon A. Marin Soto, Sonja Zweegman, Mary H. Young, Kathryn J. Newhall, Tuna Mutis and Niels W.C.J. van de Donk

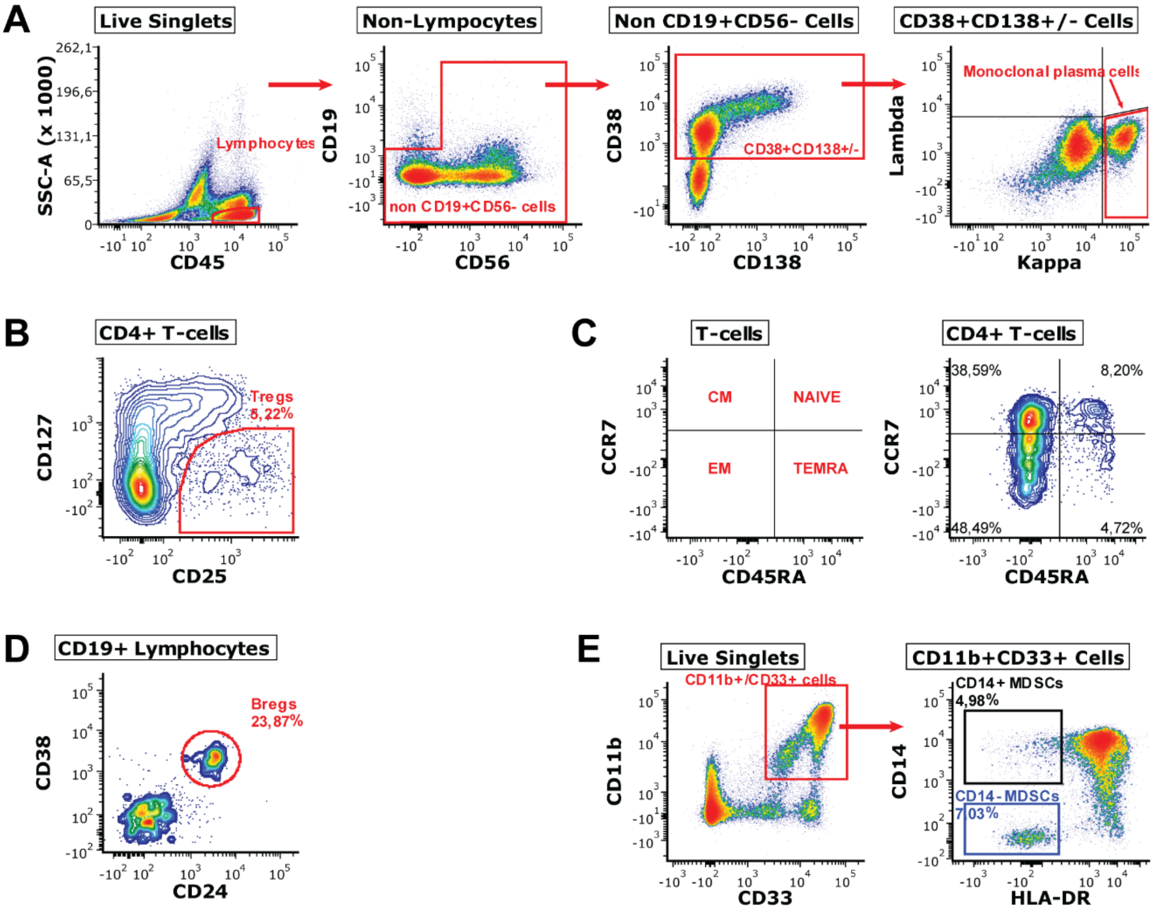

Figure S1. Gating strategy in flow cytometric analyses. Representative density or contour plots illustrate the gating strategy in a bone marrow sample of a daratumumab-refractory patient for monoclonal plasma cells (**a**); regulatory T-cells (Tregs, **b**); T-cell memory subsets (**c**) with naïve T-cells, central memory (CM), effector memory (EM), and terminally differentiated effector memory T-cells expressing CD45RA (TEMRA); regulatory B-cells (Bregs, **d**); and $CD14^+$ and $CD14^-$ myeloid derived suppressor cells (MDSCs, **e**).

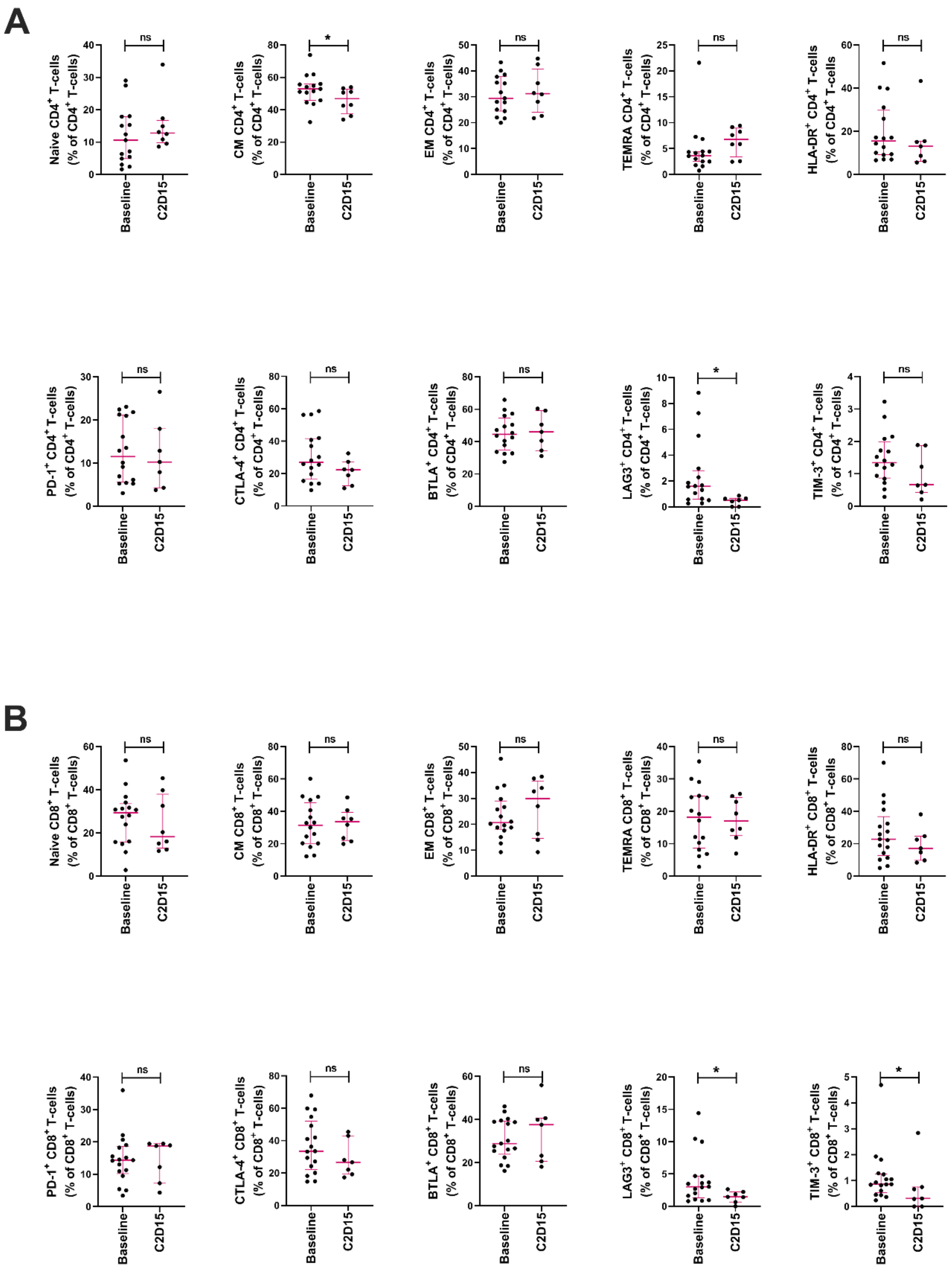

Figure S2. Effect of daratumumab and durvalumab on the frequencies and characteristics of CD4+ and CD8+ T-cell subsets. The frequency of CD4+ **(a)** and CD8+ **(b)** T-cell subsets in bone marrow samples: naïve T-cells, CM T-cells, EM T-cells, TEMRA T-cells, HLA-DR+ T-cells, PD-1+ T-cells, CTLA-4+ T-cells, BTLA+ T-cells, LAG3+ T-cells and TIM-3+ T-cells, analyzed by flow cytometry. Dots represent individual data, lines represent value and error bars represent interquartile range. Differences between baseline (n = 17) and cycle 2 day 15 (C2D15, n = 8) were assessed using Mann-Whitney U tests; *P<0.05; ns, not significant. Abbreviations: CM, central memory; EM, effector memory; TEMRA, terminally differentiated effector memory T-cells expressing CD45RA; HLA-DR, human leukocyte antigen DR isotype; PD-1; programmed death receptor 1; CTLA-4, cytotoxic T-lymphocyte associated

protein 4; BTLA, B- and T-lymphocyte attenuator; LAG3, lymphocyte-activation gene 3; TIM-3, T-cell immunoglobulin and mucin domain 3.
